# Hospital and Institutional News

**Published:** 1918-02-23

**Authors:** 


### HOSPITAL AND INSTITUTIONAL NEWS.

### THE PRINCE OF WALES AND LIMBLESS SAILORS AND SOLDIERS.

H.R.H. the Prince of Wales, who is at present home on leave, formally opened the Prince of Wales' Hospital for Limbless Sailors and Soldiers, Cardiff, on Wednesday, February 20. His Royal Highness's visit was of a semi-private nature, and the opening ceremony of a simple character, in accordance with His Royal Highness's expressed wish. The hospital was opened for the reception of patients in May 1917, and over 300 artificial limbs have already been fitted. The formal opening was delayed, however, in the hope that the Prince of Wales would be able to visit Cardiff for the purpose, seeing that the hospital had been named after His Royal Highness with his express permission. An interesting feature of the visit will be the presentation to His Royal Highness by the donors of cheques for one thousand guineas or upwards for the Endowment Fund of £100,000 to establish the hospital permanently as a memorial of the war, and to enable its benefits to be extended to civilian limbless cases throughout the Principality and Monmouthshire as soon as the demands arising from the war permit.

### "THE MODERN HOSPITAL."

The January number of *The Modern Hospital* is fuller than usual of variety, and some of the articles will prove instructive and interesting to British readers. Amongst the contents is a plan of the new Children's Hospital at Denver, Colorado, by Maurice B. Biscoe, architect, Denver, and Dr. S. S. Goldwater, consultant, New York. It presents features which cannot fail to be of interest to all concerned with hospital construction, and affords evidence of much careful thought, fulness of technical knowledge, and a useful attempt to provide for paying patients and general patients in the same institution under conditions which give the entire second floor for the accommodation of private cases in separate rooms, in addition to six private rooms on the first floor. The general plan differs from those favoured in more temperate climates, and, though not accompanied by a scale, it demonstrates that the hospital is one to be seen by hospital experts who visit the United States. Dr. Goldwater has laid himself out for a number of years thoroughly to master hospital construction. The system upon which he arranges his work, and the thoroughness with which he faces the problems of every site and the requirements of every institution, to the planning of which he gives his name and abilities, are models of the highest type of work, and the best evidence of the justice of American opinion, which has put him in the forefront, if not even, as we hold, at the head of U.S.A. hospital expert consultants who devote their attention to modern hospital construction in the best meaning of the term. His thorough conscientiousness and excellent system constitute an example to be followed.

### A STEADY DEVELOPMENT.

Dr. John A. Hornsby, the able Editor of *The Modern Hospital,* is doing most useful service through a series of articles on the Standardisation of Hospitals, the current number being devoted to Class 7, Small Municipal Hospitals. Dr. Harold C. Goodwin, M.D., has an excellent article on "The Hospital: A Teaching Institution," from which we hope to give an extract embodying the opinions of some leading medical superintendents, and the deans of medical schools in the United States, which sum up the benefits derived from a teaching hospital which cannot be had in a non-teaching medical institution. In succeeding numbers a new department devoted to war-time institutional economies is promised. It is proposed to publish the methods inaugurated in American hospitals for reducing expenses and improving the service, with directions where practical information, resting upon actual systems and experience, is available. Such information is sure of a warm welcome throughout the English-speaking world. Nurses' Homes is another interesting subject, which includes a consideration of the plans and construction of these establishments, and will, we hope, deal with their administration, and criticise and make plain in the public and professional interests the factors which have made some homes too frequently open to grave objection and even condemnation. Hospital Economy, the Advantages of State-Wide Control of Tuberculosis, the Department of Nursing and Dietetics, and much other matter have a practical value, and the high standard of literary merit maintained throughout is specially welcome. *The Modern Hospital* has developed steadily since its establishment in September 1913. We suggest, if we may, that it would be helpful to its contemporaries, and encouraging to its readers, to add the date of its first number and subsequent volumes, and occasionally to publish, if space permits, a list of previous articles which have an authoritative and permanent value.

### THE NEW CHIEF OF THE PAVILION HOSPITAL, BRIGHTON.

We understand that Colonel Thelwall Maurice, C.M.G., has been appointed officer in command of the Pavilion Hospital, Brighton. This officer has had his full share of foreign service, having been abroad practically without intermission from the early days of the retreat from Mons till he received his first leave in England a month ago. He left France for the East, was stayed at Malta to organise and equip a tent hospital there of 3,000 beds, and after completing this big task was sent to Salonika as Assistant Director of Medical Service. About a year ago his return home was interrupted by an appointment in Egypt, from which he went to Palestine and followed the British Army to Jerusalem. Shortly afterwards he returned to England, and his three weeks' leave has ended in his appointment to command the Pavilion Hospital, where, we understand, he enters on his duties this week.

### INSTITUTIONS AND THE NEW RATIONS.

Institutional caterers will now be busily studying their responsibilities under the new rationing scheme for meat, margarine, and butter. They will learn that in respect of persons resident before February 25, who take all their meals within the institution, the matter is simple, for they will be allowed to obtain supplies on order forms, taken from official order-books which will be supplied by the local Food Office, and will be specially exempted from accounting for their purchases of meat by the production of meat coupons. They will, however, be required to comply with the scale of consumption laid down by the Food Controller as appropriate to their class, and to keep records of their consumption in proof of such compliance. But in respect of persons becoming resident on or after February 25, the responsible head of a hospital or similar institution must ask for the deposit at the hospital of any food card issued in respect of a new arrival, whether a patient or a member of the staff. In respect of a non-resident taking a meal or meals at the hospital it will be necessary to depute somebody to mark off his or her card. He will also similarly mark the cards of residents who bring the cards to the hospital that have been issued at their previous residence. But what happens to a resident who wishes to obtain a meal out of the hospital or (which is a more serious difficulty) who goes away on a holiday? The public are, however, asked to bear in mind that the scheme is being put in force as a matter of urgency, and that it cannot be expected to work smoothly until there has been an opportunity of adjusting the machinery of supply to requirements.

### THE ENDOWMENT OF RESEARCH.

Few examples of public-spirited munificence can be cited that will bear comparison with that of the late Dr. Ludwig Mond, F.R.S., whose trustees have just paid over a sum of £62,000 by way of endowment to the laboratory which he presented to the trustees of the Royal Institution in 1894. By the terms of the agreement under which Dr. Mond conveyed to the institute a freehold house situated next door to their premises in Albemarle Street and bore the whole expense of fitting it out as a laboratory for chemical and physical research, he undertook to pay to them the interest upon the above-named sum for a period of thirty years, when the capital sum would be transferred to them. By thus generously anticipating their obligations by six years his trustees have materially increased the income available for the advancement of original research in chemistry and its allied subjects at a time when, in view of our exceptional economic condition, such work will have a greatly enhanced value.

### THE RELEASE OF MEDICAL STUDENTS.

The practice of allowing medical students serving in the combatant ranks to return to their studies is one which has been followed more or less regularly for some time past. But an Army Council Instruction issued a few days ago places the matter upon a clearly defined basis. "A medical student now serving with the Colours who (a) is a third-year medical student, and was at the time of his enlistment actively engaged in medical studies; or (b) at the time of his enlistment has passed the whole of the professional examination in chemistry, physics, and biology . . . and was at the time of his enlistment actively engaged in medical studies is, if he so desires, to be transferred to Class W of the Reserve." In passing, we may note the needless complication involved in forming two categories of one class, since obviously a student in (a) class is also in (b). Nevertheless, to have their duty thus clearly defined must remove any cause for heart searchings on the part of the few medical students left with the combatant forces.

### THE YEAR'S WORK AT CARDIFF.

A milestone of much interest has been reached in the history of King Edward VII.'s Hospital, Cardiff. During its existence of over eighty years—the hospital was founded in 1837—there has been a regular excess of expenditure over income year by year, amounting in the gross to £42,268. In the past fifteen years in consequence of the increased number of beds to maintain and developments in the pathological, electrical, massage, gynæcological, and hydro-therapeutic departments this gulf between expenditure and income has shown a tendency to widen. In 1917, however, as the result of a great campaign in which Colonel E. M. Bruce-Vaughan and Mr. Leonard D. Rea, the hospital's superintendent, worked like Trojans, and in which the widest possible appeal was made to popular sympathies, the year closed with an excess of income over expenditure. The whirlwind campaign was only started seventeen weeks before the end of the year, so the result is all the more a triumph to those concerned in it. The financial statement for 1917 was presented to a meeting of the management committee held on February 13, and showed that the ordinary income for the year amounted to £30,462, compared with £18,484 in 1916, an increase of £11,967. The expenditure totalled £27,612, against £23,243, an increase of £4,368, the excess of total income over expenditure for the year being £2,850.

### WHAT REMAINS TO BE DONE.

Colonel E. M. Bruce-Vaughan sent a telegram to the meeting congratulating the board on this fine achievement. He pointed out, however, that much remained undone and that they were not weary of the task in front of them. The principal task before the board, our readers may again be reminded, is the extension of the hospital, so that "that most melancholy of all queues" (as Mr. Rea calls it), the waiting list, may be dealt with. The amount required, £80,000, is only about £2 per head of the population of Cardiff, and in a city so wealthy and so generous to charitable causes, King Edward VII.'s Hospital should not long be denied the aid it seeks.

### EXTENSION PROBLEMS: TWO POINTS OF VIEW.

The recent decision to extend the Red Cross Section of the Royal Hampshire County Hospital will provide accommodation for seventy more military patients. The money required has been guaranteed, and the plans have been prepared by Lieutenant Clark, R.E., an architect who has designed many huts in the Southern Command. The advantages are not merely that more patients can be accommodated, but that by being collected into one place the time of the medical staff is saved, which, in view of the shortage of doctors, is important. This point was lately emphasised by Dr. Godwin at Winchester, who declared that even in London much time was lost through the establishment of so many different hospitals. On the other hand, there is a limit to the advantages of centralisation, and mammoth hospitals have never justified themselves, though they may become a necessity in war-time. The above scheme has had one critic in Mr. Keyser, who fears that the new extension will overstrain the administration, and his warning has at least the value that the time of the medical staff is not the only consideration.

### WOMEN COLLECTORS AT GRAVESEND.

The authorities of the Gravesend Hospital have made a feature of Christmas gift collections, and a review of seven years' effort shows that some £2,000 has been raised by this means. Its success has depended largely upon the loyal co-operation of the women friends of the hospital, and also, till paper difficulties intervened, on an ingenious series of picture appeals. In the latest collection our women took part and visited about 130 houses each. Rather more than half of the total number of houses visited—namely, 13,658—responded to the appeal, and in the end, at a cost of £21, the sum of nearly £500 was secured. It is interesting to note that not only has this success been a record, but while the number of houses visited was somewhat smaller than before, the response was fortunately greater. This is a gratifying result for Mr. C. E. Chapman, the acting secretary, and shows what can be done by persistently pursuing a method till the whole district comes to take a pride in its success.

### THE FIRST ANNUAL REPORTS.

The first annual reports with full statistical detail and complete accounts in correct form, duly audited, to reach us are those of Manchester Royal Infirmary and Blackburn and East Lancashire Royal Infirmary. We place Manchester first because the tables of patients and averages are more complete, but we congratulate both institutions on productions which say a good deal for office efficiency and administration. We understand that the Manchester report was sent to the trustees in the last days of January, and the Blackburn report was passed by the governors at a meeting held on the 14th inst. Why London should lag behind Lancashire in these matters we do not know, but metropolitan secretaries clearly will have to look to their laurels. Another remarkable achievement is the report of Pontypool and District Hospital. It is complete in every detail, including full lists of subscribers; not merely the preliminary report and accounts, such as the two mentioned above. It is on technical grounds only that it is not awarded the prize in the race for special mention, as the income and expenditure account is not quite in accordance with latest practice. But that is a matter which can quickly be put right, and Mr. T. Morgan, the secretary, must be congratulated on a production the work entailed in the preparation of which is known only to those who are engaged in hospital administrative duties. We may have something further to say about these reports at a later date, as they are worthy of more than passing notice.

### THE DISABLED MEN'S VIEW OF TRAINING.

At a recent meeting of the joint advisory committee for the Home Counties formed to deal with the question of the training of discharged disabled men, Mr. Parrick discussed the reluctance of the men to undergo the necessary "education." He said it was purely an economic question, and that the weekly maintenance allowance of 27s. 6d. during training was not attractive to men who could earn from three to six guineas a week in the dockyard. The men sent by the Government to different parts of the country were allowed £1 a week for maintenance, in addition to big wages, and he argued that disabled men should have a subsistence allowance in addition to their 27s. 6d. a week. Even if the bonus of 5s. a week promised to men at the end of their training, if this proved satisfactory, were added immediately to their maintenance money it would be an improvement. Other speakers feared that the adoption of such suggestions would open up the whole question of pensions once more, and it was decided merely to inform the Minister of Pensions of the inadequacy of the "attraction" offered. Lady Maxse's suggestion that hostels should be provided where the men would not be robbed was favourably considered.

### TREATMENT OF TUBERCULOSIS PATIENTS.

In a lecture delivered last week at the Royal Institute of Public Health Sir Arthur Newsholme emphasised the urgent need for increased sanatorium accommodation. In 1914 there were in this country 8,120 beds for adult tuberculosis patients; last year the figure had risen to 10,600. But such a number was totally inadequate for the increasing needs of the population, since a very large number of soldiers had been discharged from the Army suffering from consumption, and these naturally had the first claim. Building operations had been almost entirely stopped by the war, and until the declaration of peace there seemed to be little prospect of materially increasing the accommodation available. The education of the consumptive was a most vital question, for by training him to live hygienically the duration of his stay in the sanatorium could be appreciably shortened. Farm colonies were, Sir Arthur Newsholme suggested, an admirable solution of the problem.

### A GENEROUS PROVINCIAL PRESIDENT.

After holding the office for four years, Sir Clifford Cory, Bart., M.P., has resigned the presidency of the West Cornwall Dispensary and Infirmary at Penzance. He thinks it a mistaken policy for the office to remain in the same hands continuously, and he resigns the post after having been a generous friend to the hospital. He has, in fact, almost created a precedent for paying off debts on the year's working, having set the balance-sheet right for four years running. Besides these timely gifts he has presented the theatre with a new set of instruments and a heating apparatus, and has improved the $x$-ray installation. His successor in the presidency is Mrs. Bolitho, who, thanks to Sir Clifford's parting gift, enters office without any deficit on the funds.

### THE PROBLEM OF THE MENTALLY DEFECTIVE.

The meeting held at the Guildhall early in this month, under the ægis of the Lord Mayor, should be instrumental in arousing interest in a subject hitherto somewhat neglected by our administrators. One notices that the name of Dr. Caldecott, the able superintendent of Earlswood Asylum, is absent from the list of those present, and such an omission is the more to be wondered at inasmuch as he is one of the few who have made a special study of the defective child in its relation to the community. The training of such children with a view to rendering them self-supporting, though they may never leave the institution which shelters them, is one to which the war has lent a special urgency. Tailoring, bootmaking, basket-work, and weaving are all occupations which are readily learnt, and under proper supervision colonies can be established upon a financially sound basis.

### THE TUBERCULOSIS TRUST IN SCOTLAND.

Now in its fourth year, the Royal Victoria Hospital Tuberculosis Trust, whose headquarters are at Castle Street, Edinburgh, has fulfilled one of its objects in assisting in the foundation of a Chair of Tuberculosis at Edinburgh University. In addition, a provisional arrangement has been made to give courses of lectures on the subject to nurses. An educational campaign has been planned to take place after the war, and the cinematograph has already been enlisted to illustrate the causes of the disease and the methods of preventing it. The films thus made have been shown in the Y.M.C.A. huts at the different units, and even on the ships of the Grand Fleet. The work of the Insurance Act has been supplemented by the Trust through grants to patients without the scope of the Act in need of sanatorium treatment. Moreover, the source of infection is tackled in those families which possess a patient who has not been taken to a sanatorium by a grant to the family to enable it to leave a two-roomed house for one with larger accommodation. This reminds us once again that the disease is one of the products of poverty, and that the way to prevent its prevalence is to maintain a standard of wages which will enable people to be decently housed.

### ST. THOMAS'S HOSPITAL SPEECH CLINIC.

The Speech Clinic at St. Thomas's Hospital was established in March 1914, and may now be considered to have passed out of the experimental stage. It is worked under the direction of Dr. Charles Box, the physician in charge of the out-patient children's department, and is managed by Miss Elsie Foggerty and her pupils. It is linked up with all the other departments of the hospital, and has proved beyond doubt that its value is greatly enhanced by this connection. There are generally about fifty children attending the classes, and they are divided into seven groups:—(1) Senior boys, (2) Junior boys, (3) Girls (stammerers); (4) Lispers; (5) Chest cases; (6) Mentally deficient in speech only; (7) Cleft palate cases. Among these have been several cases of raid-shocked children completely dumb, who have recovered through relaxation and re-educative exercises. Some interesting cases of voice and speech failure after severe internal operations, or after an accident, have given excellent results. Among the group of lispers are some cases of a form of lisping or lalling due to extreme nervous tension. They yield to very much the same form of treatment as stammering, but are among the most difficult of all to reach. Ordinary speech exercises only make matters worse. A group of little people who have never learnt to speak, despite ingenious efforts on the part of their families, begin to improve almost at once, and very soon take a keen interest in mothering the latest arrival and starting it on the path of progress. In one or two bad cases of cleft palate small children have been so discouraged by their failure to articulate clearly that they remain practically speechless, and grow awkward and sulky when they are corrected. These require an infinite amount of gentle and patient treatment. No one who has watched Miss Foggerty at work can fail to realise how much of the success of the work is due to the personality and devotion of Miss Foggerty herself.

### THIS WEEK'S DRUG MARKET.

The shortage of supplies is becoming more acute, and consequently the difficulties in the way of business are increasing. Makers are unable to book orders for a growing list of drugs, and can only accept moderate orders for many others. Prompt deliveries, however, can be made of bismuth preparations, potassium iodide, corrosive sublimate, and various other commonly prescribed products. Honey has again advanced substantially in price. Saccharin is dearer. Business has been done in calumba root at a further advance in price. The value of senna is not quite so firmly maintained. Sarsaparilla is dearer. Among the articles that are becoming more difficult to buy are hexamine, potassium bicarbonate, acetanilide, tartarated antimony, bromides, and camphor. Makers of mercury ointment can only accept orders subject to their being allotted lard by the Food Controller. There are delays in the delivery by makers of ethers, caffeine, Epsom salts, sodium sulphate, and heavy carbonate of magnesia.

